# Detecting Dissonance in Clinical and Research Workflow for Translational Psychiatric Registries

**DOI:** 10.1371/journal.pone.0075167

**Published:** 2013-09-20

**Authors:** Luciana Cofiel, Débora U. Bassi, Ryan Kumar Ray, Ricardo Pietrobon, Helena Brentani

**Affiliations:** 1 Department of Psychiatry, University of São Paulo Medical School, São Paulo, São Paulo, Brazil; 2 Department of Management, Policy and Community Health of the University of Texas School of Public Health, Houston, Texas, United States of America; 3 Department of Surgery, Duke University Medical Center, Durham, North Carolina, United States of America; Maastricht University Medical Centre, Netherlands

## Abstract

**Background:**

The interplay between the workflow for clinical tasks and research data collection is often overlooked, ultimately making it ineffective.

**Questions/purposes:**

To the best of our knowledge, no previous studies have developed standards that allow for the comparison of workflow models derived from clinical and research tasks toward the improvement of data collection processes

**Methods:**

In this study we used the term dissonance for the occurrences where there was a discord between clinical and research workflows. We developed workflow models for a translational research study in psychiatry and the clinic where its data collection was carried out. After identifying points of dissonance between clinical and research models we derived a corresponding classification system that ultimately enabled us to re-engineer the data collection workflow. We considered (1) the number of patients approached for enrollment and (2) the number of patients enrolled in the study as indicators of efficiency in research workflow. We also recorded the number of dissonances before and after the workflow modification.

**Results:**

We identified 22 episodes of dissonance across 6 dissonance categories: actor, communication, information, artifact, time, and space. We were able to eliminate 18 episodes of dissonance and increase the number of patients approached and enrolled in research study trough workflow modification.

**Conclusion:**

The classification developed in this study is useful for guiding the identification of dissonances and reveal modifications required to align the workflow of data collection and the clinical setting. The methodology described in this study can be used by researchers to standardize data collection process.

## Introduction

Inefficient clinical data collection processes can result in poor data quality, poor enrollment, and ultimately limiting the applicability of the data and study feasibility [1]. Research data collection is commonly incorporated within clinical routine activities. Although previous studies have suggested that inefficiency in data collection is closely related to the alignment with the corresponding clinical workflow [2,3], to our knowledge no existing guidelines evaluate how clinical and data collection workflows should be aligned. Moreover, standardized procedures for identifying workflow dissonances are not currently available. This gap in the literature hinders the design of case report forms (CRFs) that are adequate for the data collection workflow of prospective studies. This can be particularly harmful in studies with multiple sites where differences between workflows can exist. A better understanding of how to standardize the data collection process of the different sites can lead to more reliable data.

There are important differences between clinical and research workflows [2,4]. Clinical workflow relates to the process involved with the development of clinical activities. The focal points of the tasks in this workflow is the patient, pertinent information required to establish a diagnosis and a determination of a prognosis and treatment plan for the patient, whereas in the research workflow necessary information to answer specific research questions is sought. Additionally, it might be necessary in research workflow to register information in a structured or standardized format that can be different from the usual unstructured free-text format that is commonly preferred by physicians to register clinical information [5]. These can determine the use of different instruments and forms for data collection and different processes in a workflow.

In a previous study conducted by our group [2], through a *time and motion analysis* we demonstrated that trial sites that had significant discrepancies between clinical and research workflows may present inaccuracies regarding data collection. There are several workflow analysis techniques that are useful for workflow management [6–10]. However, to our knowledge formal workflow modeling or attempts to minimize any dissonance between clinical and research workflow are rarely conducted. Alluding to musical terminology, a dissonance is defined as the "lack of harmony among musical notes” [11]. In our study we used the term dissonance for the occurrences where there was a discord between clinical and research workflows.

Workflow analysis was used to investigate and improve workflow in both clinical and research settings. In the clinical setting, the *Lean method* was used for process improvement to streamline time-dependent stroke care [12], and a *time and motion* analysis was used at outpatient pharmacies to identify opportunities to optimize the dispensing process [13]. In the research setting, the *time and motion workflow* analysis was used to provide an understanding of research workflow and identify areas where informatics-based interventions could be used to support clinical research workflow [14]. Previous studies have shown that the integration of registries into clinical workflow are vital for successful registry data collection [15], and that workflow modifications can lead to increased data collection [16].

The objective of our study was to map, record, and compare the clinical and research workflows in a psychiatric outpatient clinic. As a result of this comparison, we identified workflow dissonances and ultimately developed a workflow dissonance classification system. As a case study we implemented a series of modifications to the workflows and observed changes in patient screening and enrollment.

## Methods

This study was approved by the Institutional Review Board of the Hospital das Clínicas at the University of São Paulo Medical School in São Paulo, Brazil. The researcher explained to the patients and clinic staff (healthcare professionals involved in patient care or research data collection) that no personal information would be collected and only information regarding the clinical workflow would be registered. We received verbal informed consent from the subjects, which were obtained under supervision of the clinic coordinator and prior to data collection. The use of verbal informed consent was approved by our IRB since the data collected was related to workflow evaluation, and not patient-specific information. A proper *time and motion study* was not performed, however the workflow modeling methodology used for this case study adhered to the international reporting guidelines from a biomedical informatics perspective; detail of the methodology can be found below [17]. This study was performed from September 2010 until November 2011.

### Research Design

We performed an uncontrolled interventional study design. Field observations and interviews were performed from September 2010 until October 2010. Initially field observations with clinicians, nurses, and other staff were conducted during the first two weeks of September 2010. During this time a total of 10 hours of observations took place across two separate sessions. In the following weeks the researcher made visits to the clinic to interview the clinic staff and obtain more specific information. We created Unified Modeling Language (UML) activity Diagrams of the clinical and research workflows based on the information retrieved.

We then developed workflow analyses from November 2010 to May 2011, using a *Lean methodology* to identify workflow dissonance that led to the proposition of a new dissonance classification system; subsequently we proposed and implemented workflow modifications in order to address the identified dissonances. This new workflow was implemented in June 2011. Finally we observed the number of remaining dissonances, screened and enrolled patients from September 2010 until November 2011.

### Intervention

In the context of this case study our intervention refers to the restructuring of workflow based on the identification of dissonances between research tasks and clinical practice. Intervention was carried out in four phases: (1) research and clinical task observations and workflow modeling, (2) dissonance identification through workflow analysis, (3) the development of a workflow modification plan, and (4) the implementation of those changes as outlined in this case study.

### Observation of Research and Clinical Tasks

Workflows were initially assessed through field observations followed by interviews with doctors, nurses and the research team to confirm the collected information. Observations and interviews were conducted with the goal of understanding the major tasks and their corresponding characteristics. Although a formal *time and motion* study was not conducted, we were able to estimate the duration of the tasks based on observations and reports from the staff. Finally, we modeled the research and clinical workflow using a series of UML Activity Diagrams.

**Figure 1:**
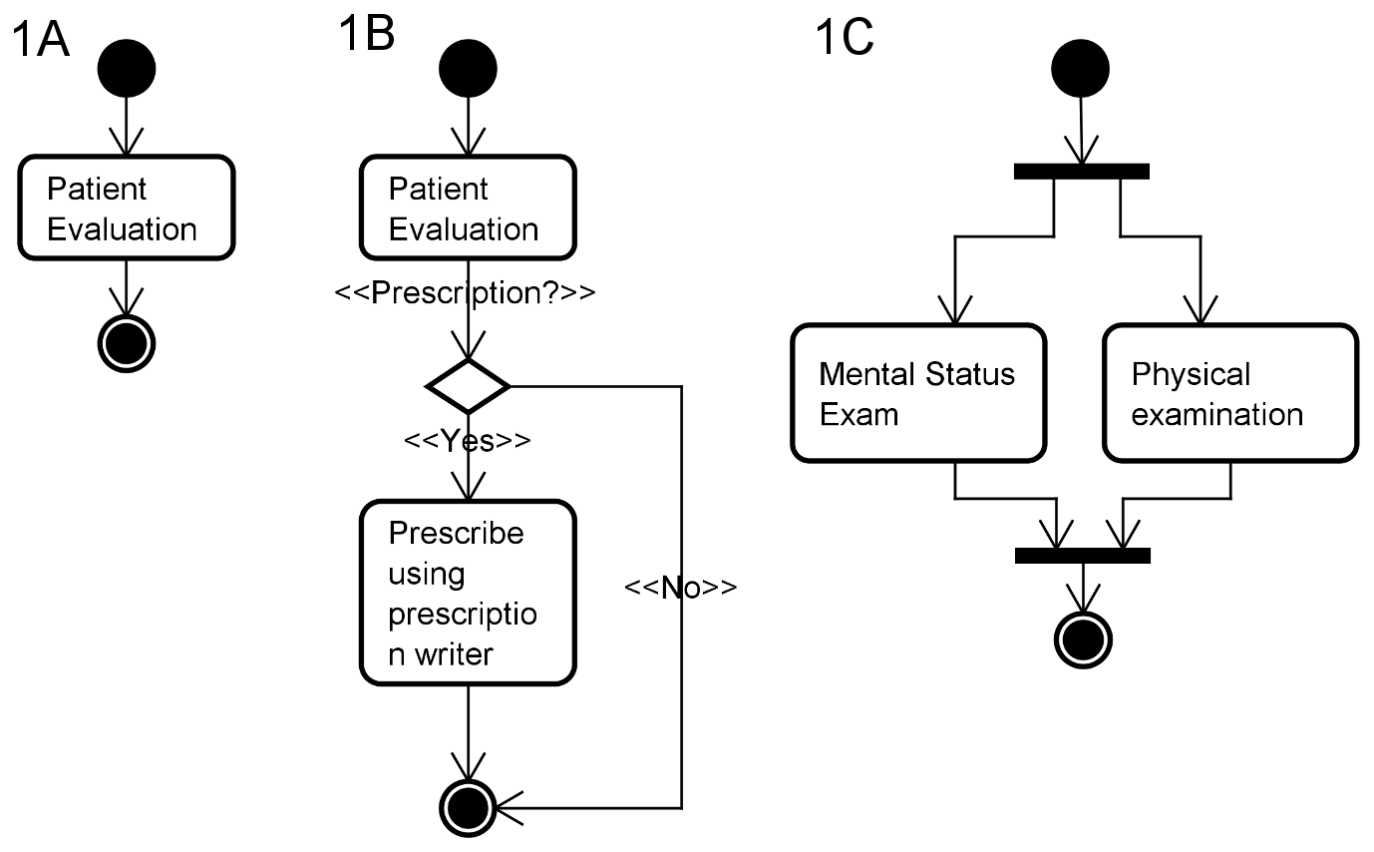
Schematic representation of the UML Activity Diagram elements. A. Initial and Final nodes, tasks and transition arrows; B. Branches and merges; C. Forks and joints.

All activity diagrams were created using Astah Professional 6.6.3 (model version 36) evaluation license [18] and were modeled according to UML version 2.0 [19]. We chose UML Activity Diagrams because they are used in sequences that model tasks with both conditional and parallel behavior [20]. In these diagrams, the starting point is indicated by a darkened circle, called *initial node*. The ending point is indicated by a darkened circle with a border, referred to as *final node*. Rounded rectangles represent *tasks*. These tasks can either be a real-world process, such as a patient assessment or the execution of a software routine, such as prescribing a medication using a prescription writer [19] (Figure 1A). Transition arrows connect the different tasks. Branches and merges are used to represent conditional behavior and are represented by diamonds in the diagram. Branches have one incoming transition and several outgoing transitions (Figure 1B). Different decisions lead to different outgoing paths. For example, when preparing a treatment plan for a patient a physician will evaluate the need for prescribing a medication. If he believes the patient should receive pharmaceutical treatment he will use the prescription writer for that task, otherwise he will take no action. Merges indicate the end of a conditional behavior that was started by a branch. Forks and joints indicate the existence of simultaneous tasks, both being represented through black bars (Figure 1C).

### Workflow Analysis for Identification of Dissonances

We defined workflow dissonances as discrepancies between research and clinical workflow. We used the *Lean method* approach for identifying workflow dissonances that generates research inefficiencies [21]. The *Lean method* is based on the Toyota model, and seeks to eliminate non-value added elements from the process, known as *waste* [21]. *Waste* includes any activity that absorbs money, time, and people but does not create value [22]. *Value* expresses the end user needs (customer) [22]. Therefore, for the evaluation of value it is necessary to determine who is the end user and what is that person’s point of view [21]. In this project we assumed the researcher was the end user, and that the *value* was the high quality data in a short period of time.

There are eight categories of activities identified as waste in the literature [22,23]. They are described as follows:

1 *Waiting* is the time spent waiting for the next process to be initiated;
2 *Overproduction*, present when the first of two sequential step processes is faster than the following step;
3 *Inventory*, which indicates products waiting for processing or to be delivered to the end user;
4 *Defects*, that demands rework for fixing problems with the product or the discard of the problematic products;
5 *Transportation* is related to the unnecessary movement of products;
6 *Overprocessing*, related to presence of processing characteristics not valued by en users;
7 *Wasted* *motion*, related to unnecessary movement of employees;
8 *Underutilized* *human* *skills and time*, present whenever a worker or equipment is idle [22,23].

The value stream defines the steps required to complete a process and the examination of the value stream reveals possible wastes within a process [21,22]. One should map the value stream and analyze it for non-value added waste and redesign the flow to remove as much of non-value added waste as possible and standardize the ongoing process. Batches and queues within a process must be eliminated so processes can flow uninterrupted and processes must be completed in order to allow the next step in the process to start [21].

We analyzed the UML Activity Diagrams to identify wastes in research and clinical workflow, and then analyzed the tasks associated with wastes using the conceptual framework, “Workflow Elements Model” proposed by Unertl and collaborators [24]. The model has both a specific and a pervasive level. The components of the specific level are the people performing actions (actors), the physical and virtual tools used (artifacts), details of actions performed (actions), characteristics describing the actions (characteristics) and the end products of the actions (outcomes). The pervasive levels (context, temporality and aggregation) have components that apply over the specific elements of workflow: the context includes physical and virtual workspace and organizational factors. The temporal factors are related to scheduling, temporal rhythms and coordination of events. Aggregation refers to the relationship and interaction among different tasks and actors [24].

We used components of the specific (actors, artifacts) and pervasive level (duration, location, period of the day) from this framework to further describe the tasks associated with wastes. Information relating to identified wastes (type and description) and task description were taken into account. We chose to describe the tasks in a broad way since this was an initial assessment of the workflow. In subsequent phases of workflow evaluation a more refined granularity could be used to reveal issues related to the tasks that a more general analysis was not able to show. We also registered the data variables generated at each task as this indicates that the task is of particular importance for the end user (researcher).

We investigated which workflow component was associated to the dissonance and named it accordingly. The designation specifically alluded to the aspect of the activity that represented the source of inefficiency. Most importantly, this inefficiency indicates a flawed aspect of the integration of the research and clinical workflow. This procedure was carried out for all of the tasks. Two additional authors reviewed the process (RP and HB).

### Development of Workflow Modification Plan

Workflow modifications were focused on the elimination of the most frequent dissonances and then the remaining dissonances by decreasing frequency. Strategies for improving the overall clinical workflow while allowing more efficient data collection was discussed with the clinic coordinator. As a result, we created a new workflow addressing the dissonances that were identified based on these analyses and group discussions with the clinic coordinator.

### Implementation of Changes

As a case study, we implemented the proposed workflow based on the dissonance analysis. After the clinic director approved the proposed workflow it was presented by the clinical coordinator to professionals from the outpatient clinic during a staff meeting. At that time the clinic staff and researchers were trained to use the new tools so that they would be able to perform the new tasks. One week later, the clinic implemented the new workflow. All workflow modifications were implemented simultaneously in the first week of June 2011.

**Figure 2:**
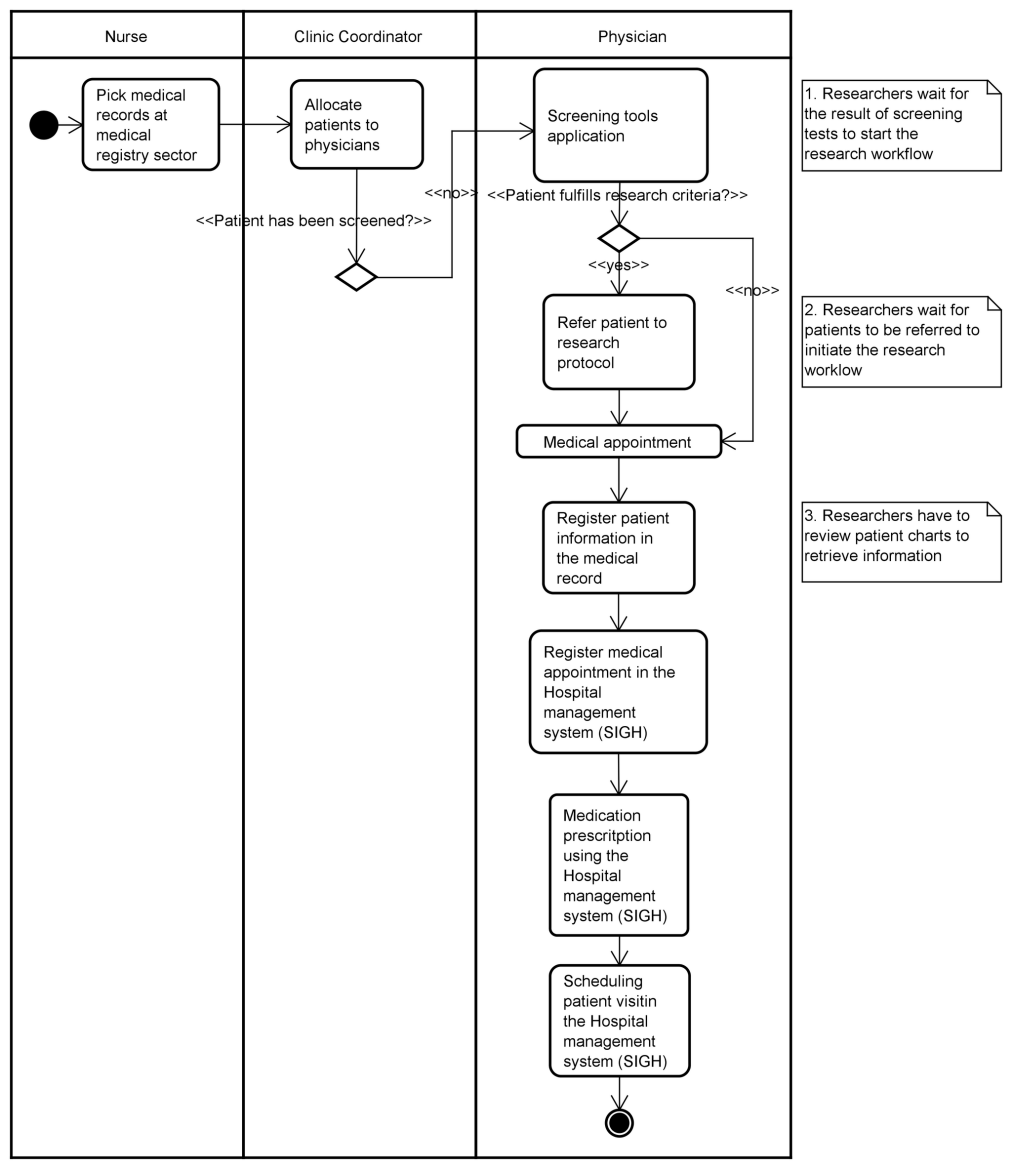
UML representation of the identified for the clinical workflow.

We considered the following measures as indicators of efficiency in research workflow: (1) Number of patients approached for enrollment, (2) number of patients enrolled in the study. This information was derived from a spreadsheet that was used to register information about the patients who were screened, contained patient names, identification numbers, screening assessments dates, screening assessment results, and information on whether the patients had been included in the research protocol. We obtained data corresponding to the period between September 2010 and November 2011. December 2010, January and July 2011 were not included since the outpatient clinic was closed. We also recorded the number of dissonances before and after the workflow modification.

**Figure 3:**
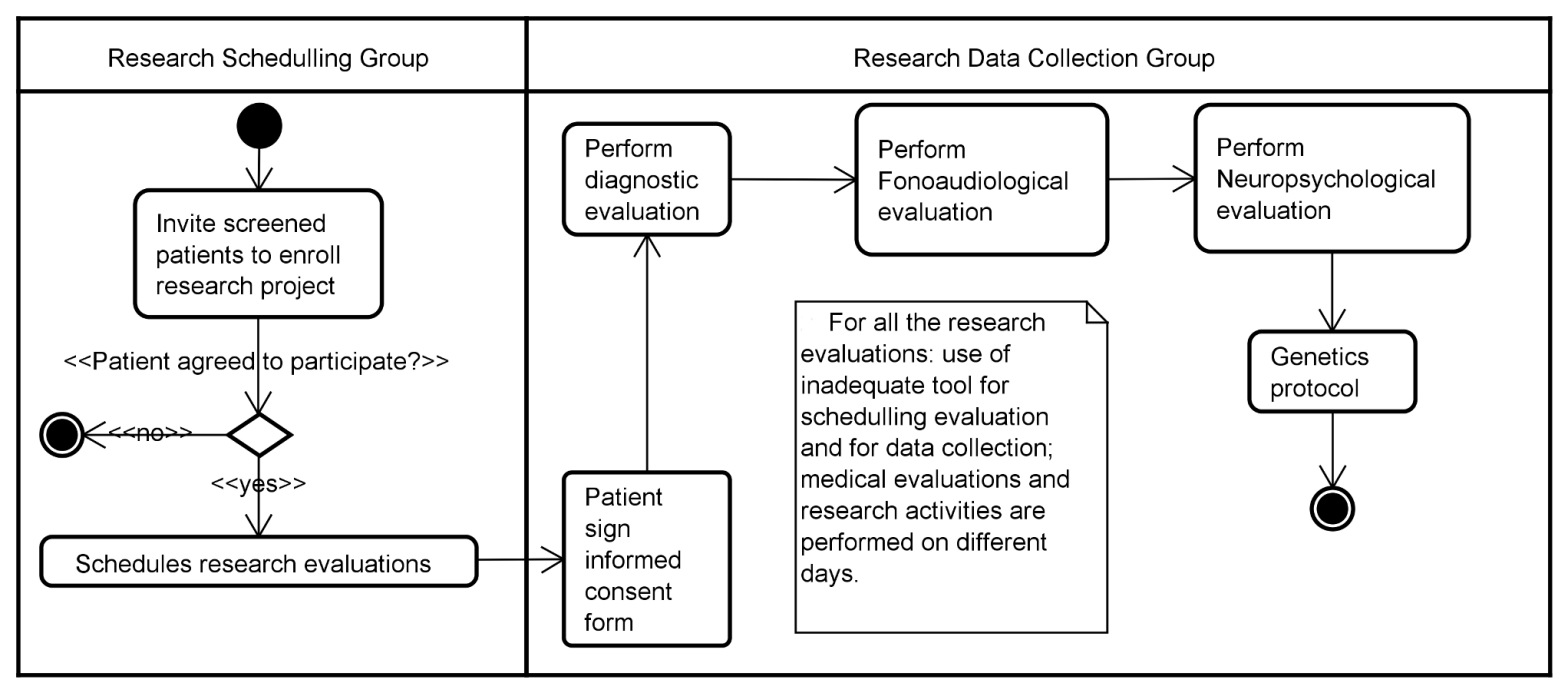
UML representation of the identified for the research workflow.

### Empirical Setting

We conducted our observations at the Autistic Spectrum Disorder outpatient clinic at the Institute of Psychiatry at the Hospital das Clínicas, an urban teaching hospital at the University of Sao Paulo Medical School. Its mission is to integrate health care, education, and research in order to better serve the community. The clinic operates one morning a week with an average 20 patients per week. We selected this clinic for our case study as it initially seemed like a location where clinical and research workflows were misaligned. One important research objective in this clinic was the development of diagnosis assessment protocols and assessment tools. However, the research process was hindered by the limited number of patients that were screened (evaluated with autism screening instruments) and enrolled into research protocols.

### Tasks Category

The observer did not use a predefined list of tasks since the main objectives of the observation were to describe and document the overall clinic workflow. Tasks of interest included all those necessary to complete the clinical workflow (the outpatient medical visit for one patient) or the research workflow (research data collection for one patient).

### Observer

One of the authors (LC) conducted the observations and interviews. This researcher did not participate in specific training prior to data collection, but had a background in health informatics and prior experience in workflow analysis for information systems implementation.

### Subject

The clinic had a team composed of four residents, two medical assistants, and a nurse involved solely in patient care. Additionally there was a multidisciplinary team involved in research and patient care. This team was comprised of one occupational therapist, three psychologists, one neuropsychologist, and two speech therapists.

A physician and a representative of each multidisciplinary team member category selected by convenience were accompanied during one morning (from 8 am to 11 am) so the observer could understand the routine pattern of their tasks. The remainder of the observation period was used to observe the general approach and interaction of researchers towards the patients, and the interaction among the clinic staff.

### Data Recording and Analysis

Observational data was recorded through notes collected by the author (LC) during fieldwork and a qualitative approach was used to analyze this data.

## Results

### Clinical and Research UML Workflow Modeling Results

Two distinct activity diagram workflows present the main tasks identified during the observation period. The Extensible Markup Language (XML) Metadata Interchange (XMI) models from all of the UML models created in this research are available at figshare.com [25]. Models for the clinical and research workflow are presented in Figures 2 and 3.

### Observation and Dissonance Classification Results

We analyzed the UML Activity Diagrams to identify existing wastes and opportunities to integrate process steps more efficiently, and wastes were indicated as notes in the UML Activity Diagrams (Figures 2 and 3). Waste, its description and the tasks associated with the waste are presented in the first three columns of Table 1. Additionally, details of the tasks are presented in presented in the 4th through 10th columns and workflow dissonances designations are presented in the last column.

**Table 1:** List of tasks and associated wastes identified in the workflow analysis are presented in the first three columns (bolded text); detailed information regarding tasks are presented in the 4^th^ through 10^th^ columns (plain text) entitled: “Actor” (people performing the activity), “Category” (clinical or research workflow), “Duration” (estimated duration of the activity according to actors report), “Location” (physical location where the activity is carried out), “Day Period” (period of the day when the activity occurs), “Tool” (tools necessary to carry out the activity), and “Variables” (data collected through the activity aimed to collect); workflow dissonance associated to the activity is presented in the last column (text in *italic*).

**Task**	**Waste**	**Waste description**	**Actor**	**Category**	**Duration**	**Location**	**Day period**	**Artifacts**	**Variables**	***WorkflowDissonance***
**Screening tools application**	**Defect; Waiting**	**Use of inadequate tool for data collection; Researchers have to wait for the result of the screening evaluations to start research evaluations**	Physician	Clinic Routine	15 min	Outpatient clinic	Morning	Paper form	Score	*Artifact dissonance; Actor dissonance*
**Refer patient to research protocol**	**Waiting**	**Researchers have to wait physicians refer patients to the research protocol**	Physician	Research Routine	5 min	Outpatient clinic	Morning	None	Meet research inclusion/ exclusion criteria	*Information dissonance; Communication dissonance*
**Register patient information in the medical record**	**Overprocessing**	**Researchers have to review medical records to retrieve patient information**	Physician	Clinic Routine	5 min	Outpatient clinic	Morning	Patient chart (paper)	Patient clinical information	*Information dissonance; Artifact dissonance*
**Diagnostic assessment application**	**Defect; Transportation**	**Use of inadequate tool for scheduling evaluation; Use of inadequate tool for data collection; Research evaluation and medical appointment are scheduled on different days, and are performed at different locations**	Research group	Research routine	60 min	Outpatient clinic	Afternoon	Paper form	Instrument score	*Artifact dissonance; Information dissonance; Time dissonance; Space dissonance;*
**Speech language pathology assessment**	**Defect; Transportation;**	**Use of inadequate tool for scheduling evaluation; Use of inadequate tool for data collection; Research evaluation and medical appointment are scheduled on different days, and are performed at different locations**	Research group	Research routine	60 min	Outpatient clinic	Afternoon	Paper form	Speech language pathology assessment	*Artifact dissonance; Information dissonance; Time Dissonance; Space Dissonance*
**Neuropsychological assessment**	**Defect Transportation;**	**Use of inadequate tool for scheduling evaluation; Use of inadequate tool for data collection; Research evaluation and medical appointment are scheduled on different days, and are performed at different locations**	Research group	Research routine	60 min	Neuropsychological sector	Afternoon	Paper form	Neuropsychological assessment	*Artifact dissonance; Information dissonance; Time Dissonance; Space dissonance;*
**Genetics protocol**	**Defect; Transportation**	**Use of inadequate tool for scheduling evaluation; Use of inadequate tool for data collection; Research evaluation and medical appointment are scheduled on different days, and are performed at different locations**	Research group	Research routine	30 min	Outpatient clinic	Afternoon	Paper form	Genetics assessment	*Artifact dissonance; Information dissonance; Time dissonance; Space dissonance*

We observed three tasks related to the clinical workflow that when not accomplished could delay the research workflow and were associated with wastes (Figure 2).

The task, “screening tools application” was related to the waste type *waiting* since researchers would have to wait for physicians to screen patients before they could perform their evaluations. The review of the detail of these tasks indicated that the number of actors (physicians) limited the clinic’s ability to successfully conduct screening evaluations. Also, residents were assigned to the clinic for only a semester for each period, and therefore new physicians needed to be trained to use the screening tools. This inefficiency was then designated as “actor dissonance,” since the specific cause of the disruption of research workflow was directly linked to the actor of this activity.

**Figure 4:**
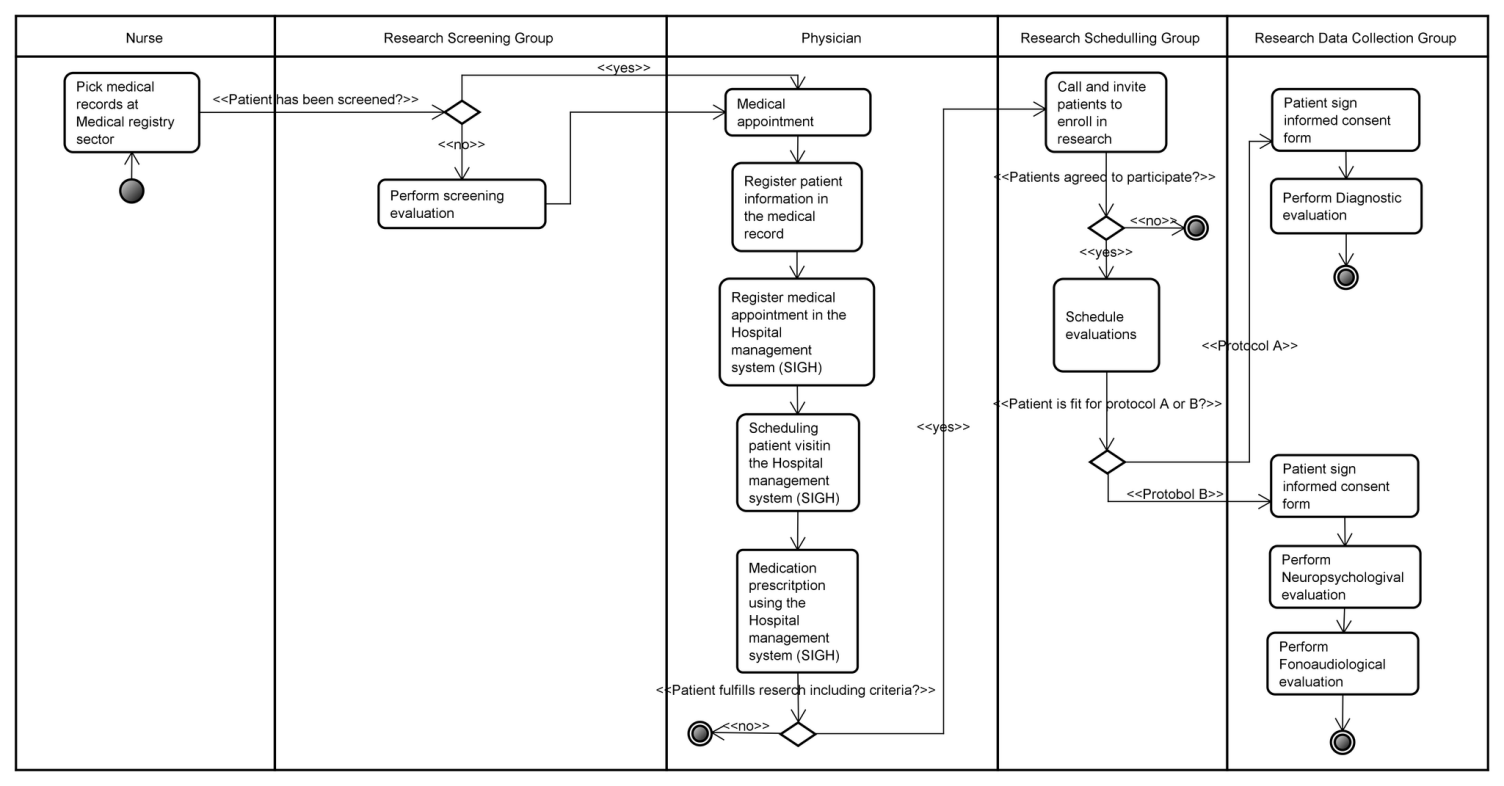
Integrated clinical and research workflow.

The use of paper-based forms for performing screening is associated with the waste type *defect* since it demands rework for the researchers as it is necessary to reenter the data in an electronic format. This strategy hindered real-time sharing of the results with researchers so it was classified as “artifact dissonance.”

The task, “referring patients to research protocols” is related to the waste type *waiting* since researchers would have to wait for physicians to refer patients to the research protocol before they could perform their evaluations. Some physicians did not know the inclusion and exclusion criteria, thus hindering the referral of potential subjects for research protocols. This was classified as “information dissonance”. Even when patients were screened and met research inclusion criteria communication problems existed between the physicians and researchers and undermined the referral of patient and this was designated as “communication dissonance.”

**Figure 5:**
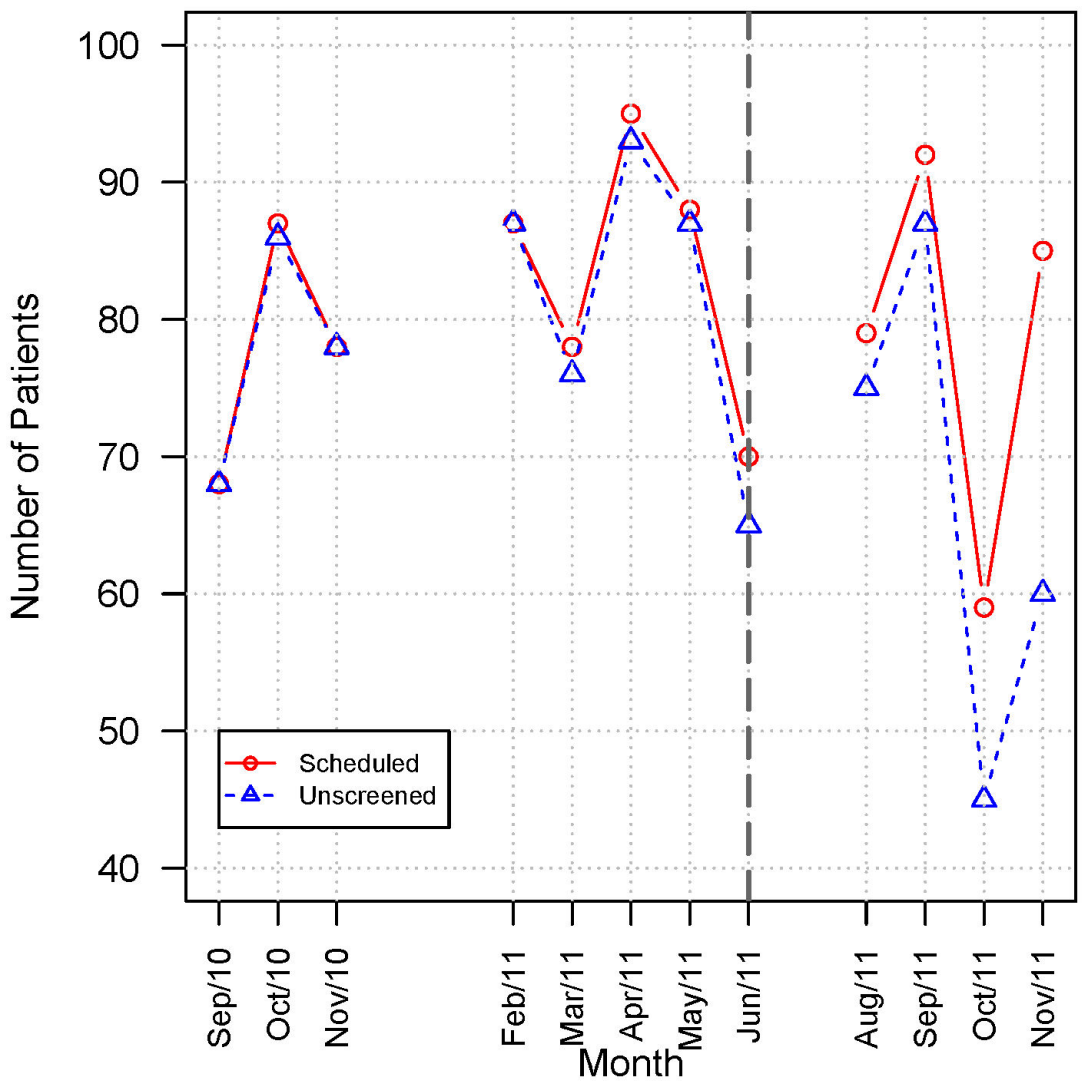
The number of medical visits pre- and post intervention at the Autism Outpatient Clinic. The number of medical visits per month and corresponding number of patients not yet screened are presented before and after intervention. Data refer to the period between October 2010 and November 2011 at the Autism outpatient clinic.

The problems with the referral process motivated researchers to look for subjects by reviewing patient charts. The task, “register patient information in the medical record” was associated with the waste type *overprocessing* since researchers had to manually review the patient chart.

The analysis of the UML Activity Diagrams indicated the existence of waste from some tasks of the research workflow (Figure 3). We observed two types of wastes associated with the tasks of performing research evaluation: *defect* and *transportation*. *Defects* were associated with the use of inadequate tools for scheduling and registering research evaluations. The use of paper-based forms to collect research data led to rework, since it was required to input collect data on a spreadsheet, and also implicated fragmented information. These were designated as “artifact dissonance” and “information dissonance.” Research evaluations were also associated with *transportation* waste since research evaluations had to be carried out at a different time and locations than where clinical tasks took place. These were designated as “time dissonance” and “space dissonance”.

**Figure 6:**
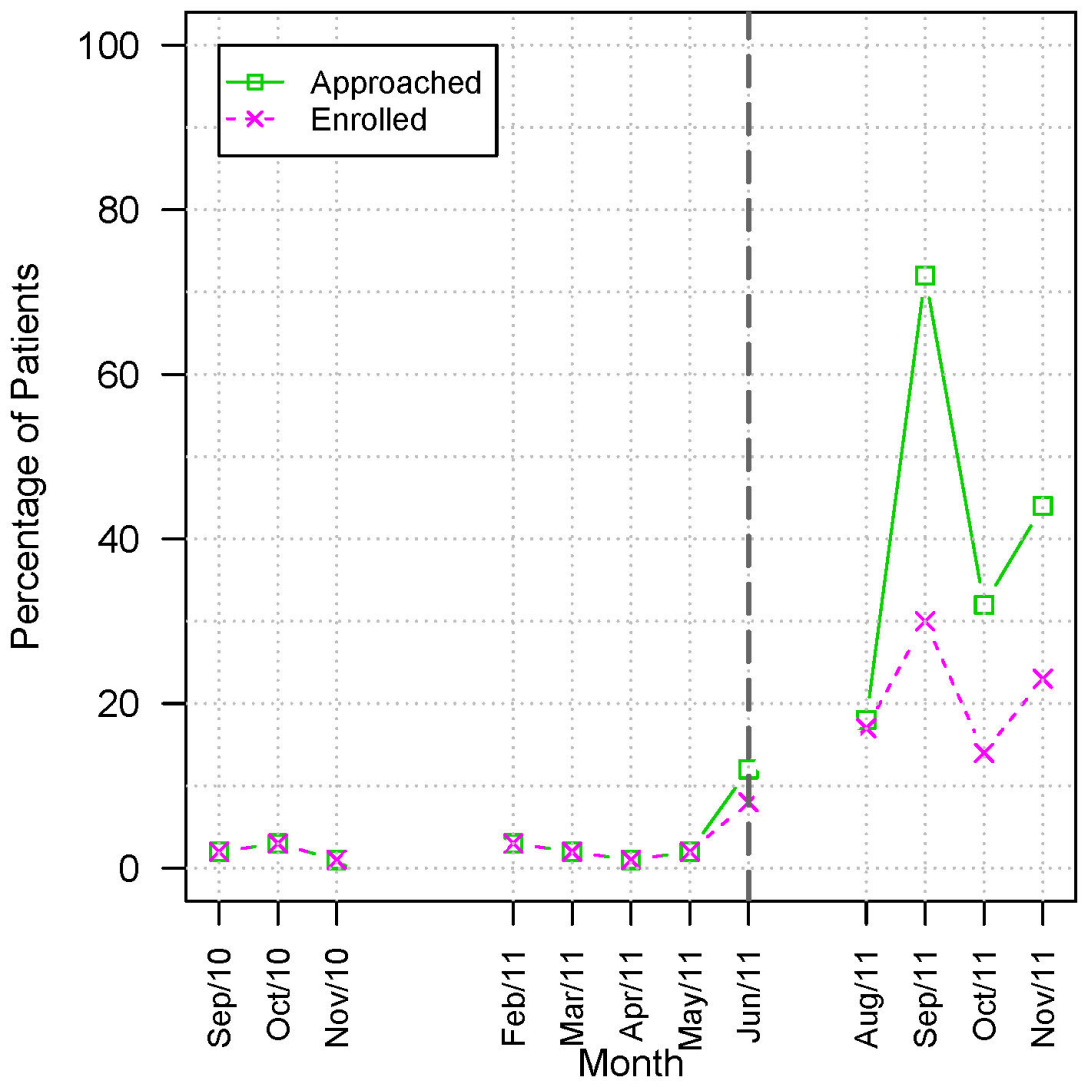
Pre and Post intervention screening and enrollment at the Autism Outpatient Clinic. Percentage of patients approached and enrolled in relation to total number of patients available for screening for the month. Data refer to the period between October 2010 and November 2011 at the Autism outpatient clinic.

Observed factors regarding tasks that disrupted workflow and corresponding classification are presented in Table 2. This list was then summarized to eliminate duplicate terms and improve the overall ease of use of the classification and presented under Table 3.

**Table 2:** Factors that impaired workflow and designated classification.

**Dissonance classification**	**Identified problems**
**Actor dissonance**	The number of actors (physicians) limited the clinic’s ability to successfully conduct patient screening. Also, the residents remained at the clinic for only 6 months, so it was always necessary to train new residents in the use of screening tools.
**Information dissonance**	Physicians were supposed to refer patients evaluated with the screening tools to the research protocol, so they had to know all the inclusion and exclusion criteria for the different protocols.
**Communication dissonance**	Ineffective communication between physicians and researchers undermined patient referral.
**Information dissonance**	The research team attempted to identify possible eligible research subjects by going over patients’ paper-based medical records. However, the use of free text to record patient care, the large volume of information within the patient record, and difficulties understanding physician handwriting hindered this process. Diagnosis and comorbidities not properly specified in the records lead the researchers (psychologists, speech therapist and occupational therapists) to recruit unsuitable subjects.
**Information dissonance**	Some researchers did not have access to the hospital management system for scheduling patient appointments. This hindered the scheduling of evaluations efficiently.
**Artifact dissonance**	Scheduling of patient medical appointments was done using the hospital management system, while the scheduling of the research assessments was done using a spreadsheet. The use of these different tools hinders the assessments scheduling process, since the researcher has to look up each patient’s upcoming appointment and then confirm that date with the researcher performing the assessment. The spreadsheet itself is inefficient because other researchers are unable to consult it, so every scheduled event must be disclosed by email. If an assessment must be rescheduled, the investigator must confer with the researcher performing that assessment in order to determine the best date for new assessment. After confirming the patient availability, he must then update the spreadsheet.
**Time dissonance**	The research team contacted patients who met inclusion criteria so that the first research assessment could be performed. However, a 30-day interval could exist between this screening and the first research assessment. Moreover, two research visits were necessary since the assessment could not be carried out on a single day.
**Space dissonance**	All rooms in the outpatient clinic were used by physicians for patient care. Research assessment could not be performed on the same day of the patient medical appointment because there was no office dedicated to carrying out research assessments.
**Artifact dissonance**	All the researchers used paper-based forms to register their assessments. Each researcher was responsible for transcribing each result to a spreadsheet. This process was error-prone because mistakes could occur while transferring information from paper to spreadsheets. In addition, paper forms cannot have automated form validation and can be lost resulting in missing data.
**Information dissonance**	Results from research assessments were kept updated on a separate spreadsheet by each researcher, and this information was not shared with the other researchers or the physicians.

**Table 3:** Final classification for the dissonance observed on the present study.

**Dissonance type**	**Definition**
Actor	Actors are not available, or are not prepared to perform a specific task
Communication	Presence of inefficient communication among actors, preventing task execution
Information	Insufficient information for executing a task
Artifact	Use of inadequate tools when executing a task
Time	Existence of time lag between tasks of clinical and research workflow
Space	Use of different physical spaces for performing tasks of clinical and research workflow

### Case Study Proposing New Workflow

Table 4 presents the frequency of the workflow dissonance observed in the initial workflow analysis. A total of 22 episodes of dissonance were observed across 6 dissonance categories. The most frequent dissonance belonged to the information and artifact dissonance categories, followed by the time and space dissonances. The least frequent dissonances were the actor and communication dissonances. We proposed five modifications to address the identified workflow dissonance points by aiming to eliminate the most frequent dissonances first:

1 We replaced the paper-based data collection system with an electronic data capture [26]. This addressed the “artifact dissonance” and “information dissonance” by eliminating rework for researchers and allowing information exchange among the clinic’s multidisciplinary team.
2 We granted the research team consultation access to the patient scheduling information in the hospital management information system. This information enabled scheduling of research evaluations more efficiently and addressed one source of “information dissonance.”
3 We reorganized the flow of activities so the time patients spent in the waiting room could be used for the application of screening tests. Patients who had a screening score compatible with the inclusion criteria for the research protocol would be informed about the study and asked to participate. As a result researchers could quickly schedule visits and decrease the number of patients who declined to enroll in the study since patients are more likely to accept when they are approached in person rather than over the phone. We also proposed scheduling research assessments on the same day they had their medical appointment. This measure was also able to address “time dissonance.”
4 All the research assessments could not be developed at the clinic at the same period as medical consultations due to limitation of physical space. We divided research tasks into two modules. The first was performed while patients were waiting before or after the medical visit. The second module, with time consuming evaluations was performed in the afternoon. This measure partially addressed the “time and space dissonance.”
5 We transferred the responsibility of performing the screening evaluations to two researchers and this addressed the “actor dissonance.” This also addressed the “communication dissonance” since physicians no longer had to refer patients for participation in the research protocol, and the “information dissonance” since researchers no longer had to review patient charts to identify potentially eligible subjects.

We designed a new workflow combining research and clinical tasks by implementing all the above mentioned modifications (Figure 4). We were not able to address the source of dissonance related to the difficulty of retrieving information from the paper-based medical record. However, there is an ongoing project of implementing an electronic medical record that will be able to address it.

### Pre and Post-Intervention Patient Enrollment

The number of medical visits per month and corresponding number of patients not yet screened are presented in Figure 5. The number of appointments per month varied each month and was influenced by the occurrence of holidays during the period. However, it did not change after the implementation of the new workflow (indicated by a dashed line). During the months of the study patients that were previously screened returned for follow-up visits, thus contributing to a decrease in the number of patients that were still available for screening.

Figure 6 presents the percentage of the patients screened and enrolled in relation to the total number of patients available for screening during the months of the study. A dashed line indicates the moment of the workflow modification. We observed a marked increase in the number of patients screened and enrolled after the implementation of the new workflow.

At the end of the observation period there was a small decrease in the percentage of patient screened. However, the percentage of patient enrollment remained steadily increasing (Figure 6). Dataset and scripts that were used to generate plots are available at Figshare [25].

Table 4 presents the frequency of the workflow dissonance remaining after the new workflow implementation. The number of dissonances decreased from 22 to 4. The “actor” and “communication dissonances” were addressed. One episode of information and one of artifact dissonance related to the use of a paper-based patient medical record remained unresolved. One time and space dissonance was not addressed, as the more time-consuming evaluations had to be carried out after the medical appointment.

## Discussion

In this study we evaluated and implemented workflow modifications related to research efficiency. We developed a classification for workflow dissonance that can be used to evaluate and adjust workflow prior to data collection.

We used UML Activity Diagrams to represent workflow, which also were used for the identification of dissonances. A variety of modeling languages are currently available for the representation of workflow models in clinical research [2,14] and patient care [27,28]. However, the lack of standards for model representation hinders the comparison across different models. UML Activity Diagrams can be used as an interoperable format for workflow modeling offering an attractive alternative to address standardization challenges [2]. This standard can facilitate the comparison among different research sites. The creation of UML models requires the effort of a trained professional in a particular modeling language. However, the use of collaborative and user-friendly tools such as Moki [29], which combines the use of formal and informal data could lead to a significant simplification in the development of workflow models.

“Artifact” and “information” were the two most frequent dissonances. “Artifact dissonance” originated from the use of paper-based tools, such as research forms and paper-based patient medical charts. This is consistent with the results of previous studies reporting the extensive use of paper-based tools in research [14], despite the fact that there are many electronic systems available [30]. However, it is worth mentioning that there are some advantages of paper-based tools over computer-based tools, including simple forms development and implementation, low utilization costs, and little to no need for support, equipment or training [1,31,32]. The use of these tools also contributed to the large number of information dissonance as these tools decreased the efficiency in tasks concerning information retrieval [14], the generation of redundant data entry, and decreased information reutilization [33].

We found that automated research data capture was beneficial in that it allowed professionals involved in both research and patient care to share information. Furthermore, the authors made the content of the questionnaires available to the clinicians. As a result, patients were more willing to enroll in the research protocol because they knew that their information would be available for physicians and used as part of their care. Previous study showed that patients are more cooperative when they know that data collected for research is also important for their treatment [16].

**Table 4:** Frequency (percentage) of workflow dissonance events identified before and after changes in the clinical and research workflow.

**Dissonance type**	**Pre-intervention frequency of dissonance events(N= 22**)** Freq(perc**)	**Post-intervention frequency of dissonance events (N=4**)** Freq(perc**)
Actor	1 (4.5)	0 (0)
Communication	1 (4.5)	0 (0)
Information	6 (27.3)	1 (25)
Artifact	6 (27.3)	1 (25)
Time	4 (18.2)	1 (25)
Space	4 (18.2)	1 (25)
**Total**	**22 (100%)**	**4 (100%)**

The “actor dissonance” was related to the fact that actors from the clinical workflow were responsible for the tasks of screening and referring patients to the research protocol. As previously observed in medical registry evaluations [15,34], the support and involvement of physicians with data collection is important for compliance, and therefore incentives might be needed to motivate the physician participation over time [34]. The information collected for research purposes could be beneficial for healthcare purposes but since physicians did not have access to the outcomes of other research evaluations performed by the multidisciplinary team they were not motivated to participate in the research workflow. This was of particular importance since this dissonance represented a bottleneck for the start of the research workflow.

“Time” and “Space dissonance” were often observed together in our study. Often patients are not willing to come to the hospital solely for the purpose of participating in research projects. Performing screening tests and less time consuming research evaluations on the same day as the medical appointment effectively led to an increase in the number of patients screened and enrolled. Besides increasing enrollment this strategy can facilitate the scheduling of research evaluations [33].

Throughout the workflow modification process we observed that one measure (such as the replacement of paper-based tools by the electronic data capture system) impacted a number of different dissonances, thus indicating that many dissonances were related. It might be impossible to separate all the complexities present in a workflow. Therefore, process improvement activities must be carried out in an incremental manner so the impact of changes can be evaluated at each implementation cycle. The evaluation will show whether a dissonance was misinterpreted or if the proposed intervention was ineffective.

A formal evaluation of data quality was beyond the scope of this work and is under evaluation in other project. After the electronic data capture system implementation data was entered directly into the system. Without a corresponding paper source we were not able to ensure that the information added was accurate. It is worth noting that the direct data entry without a paper source documentation is a standard accepted by the Food and Drug Administration (FDA) among others [35].

Despite contributing to the workflow literature in biomedical informatics our study presents limitations. A limited number of actors were interviewed during the data collection and the classification created was based only on data from one clinical and one research workflow analysis. Moreover, our study was solely focused on the evaluation of a workflow related to research registries and prospective research studies. The pre- and post study design used should be considered as a case study and our results should be interpreted as a first appointment. A proper case control study is needed to establish the association between intervention and outcome. Further, we were not able to specify individual contributions of each modification to the overall workflow improvement. The increased number of patients screened and enrolled might be confounded by the Hawthorne effect [36], which states that subjects’ behavior or study results can be modified if the subject receives additional attention or is aware that he is being investigated. A prolonged observation period is necessary to confirm the stability of the gains observed. Nevertheless, this potential efficiency gain is particularly important given the central role of enrollment to determine the success or failure of clinical studies. The difficulty in enrolling large samples of autistic patients has been reported as a barrier to the advance in the field of autism, especially in Brazil, with efficiency gains being of great importance [37,38].

Future studies are necessary to validate our methodology. Specifically, using the dissonance classification in the evaluation of studies with different research designs can provide further insights in expanding and modifying our classification. Also of importance, future studies should focus on evaluating data quality.
